# Imaging of VSOP Labeled Stem Cells in Agarose Phantoms with Susceptibility Weighted and T2* Weighted MR Imaging at 3T: Determination of the Detection Limit

**DOI:** 10.1371/journal.pone.0062644

**Published:** 2013-05-08

**Authors:** Donald Lobsien, Antje Y. Dreyer, Albrecht Stroh, Johannes Boltze, Karl-Titus Hoffmann

**Affiliations:** 1 Department of Neuroradiology, University Hospital Leipzig, Leipzig, Germany; 2 Fraunhofer-Institute for Cell Therapy and Immunology, Leipzig, Germany; 3 Translational Centre for Regenerative Medicine, University of Leipzig, Leipzig, Germany; 4 Research Group Molecular Imaging and Optogenetics, Institute for Microscopic Anatomy and Neurobiology, Focus Program Translational Neuroscience, University of Mainz, Mainz, Germany; 5 Massachusetts General Hospital and Harvard Medical School, Neuroscience Center, Charlestown, Massachusetts, United States of America; New Jersey Medical School, University of Medicine and Dentistry of New Jersey, United States of America

## Abstract

**Objectives:**

This study aimed to evaluate the detectability of stem cells labeled with very small iron oxide particles (VSOP) at 3T with susceptibility weighted (SWI) and T2* weighted imaging as a methodological basis for subsequent examinations in a large animal stroke model (sheep).

**Materials and Methods:**

We examined ovine mesenchymal stem cells labeled with VSOP in agarose layer phantoms. The experiments were performed in 2 different groups, with quantities of 0–100,000 labeled cells per layer. 15 different SWI- and T2*-weighted sequences and 3 RF coils were used. All measurements were carried out on a clinical 3T MRI. Images of Group A were analyzed by four radiologists blinded for the number of cells, and rated for detectability according to a four-step scale. Images of Group B were subject to a ROI-based analysis of signal intensities. Signal deviations of more than the 0.95 confidence interval in cell containing layers as compared to the mean of the signal intensity of non cell bearing layers were considered significant.

**Results:**

Group A: 500 or more labeled cells were judged as confidently visible when examined with a SWI-sequence with 0.15 mm slice thickness. Group B: 500 or more labeled cells showed a significant signal reduction in SWI sequences with a slice thickness of 0.25 mm. Slice thickness and cell number per layer had a significant influence on the amount of detected signal reduction.

**Conclusion:**

500 VSOP labeled stem cells could be detected with SWI imaging at 3 Tesla using an experimental design suitable for large animal models.

## Introduction

Ischemic stroke is one of the primary causes of acquired disability in adults in the western world [1]. Therapeutic options are limited. Particularly the timely recanalization of occluded vessels as the only FDA-approved therapeutic intervention so far is feasible only in a small number of patients [2]–[6]. Hence, there is a strong demand for alternative therapeutic strategies and beneficial effects could be demonstrated by administration of stem cell therapy after stroke, mainly in small animal models. However, the exact pathophysiological mechanisms and the optimal form of stem cell therapy still need to be elucidated [5], [7]–[9]. For example, it is still not clear whether a particular stem cell population is required to be present in the brain to unleash optimal therapeutic effect. This is most likely the case for some particularly promising stem cell populations thus tracking of intracerebrally located cells in the human brain will become a relevant safety endpoint [10]. Therefore, different labeling techniques are already used to track stem cells in vivo. One promising technique is the labeling of stem cells with iron oxide nanoparticles and subsequent magnetic resonance imaging (MRI) [11]–[16]. It has been shown at 7 Tesla and with T2* weighted sequences that stem cells labeled with very small superparamagnetic iron oxide particles (VSOP) migrate to the border of ischemic regions within the brains of splenectomized mice after systemic application [17]. However, a transition of these results to large animal models is desirable for several reasons such as the better differentiation of the brain anatomy with clinical MRI scanners, the higher similarity of the gyrencephalic brain anatomy to human brains, the more complex behavioral patterns and the potential of long term safety/efficacy analyses using large animal models [18], [19]. On the other hand, large animal models require larger bores of the MRI scanners, different coils and therefore use lower field strengths in most cases. This results in limitations of the achievable spatial resolution and of the detectability of labeled cells with T2* weighted imaging [20], [21]. Susceptibility weighted imaging (SWI) is an alternative to T2* weighted sequences for the detection of signal changes due to ferro- and paramagnetic effects. It has been shown that SWI may provide a higher resolution and a higher sensitivity for the imaging of ferromagnetic and paramagnetic effects than T2* weighted imaging [22]–[25]. This could be used to compensate, at least in part, the above mentioned limitations of large animal examinations.

Here, we examined the sensitivity of SWI in comparison to T2* weighted imaging for the detection of VSOP labeled mesenchymal ovine stem cells in agarose phantoms at 3 Tesla in an experimental setting suitable for the application in large animal models.

## Materials and Methods

### Ethics Statement

All animal experiments were approved by the Experimental Animal Committee of the Regional Council of Leipzig (TVV 16/07).

### Stem Cells

Autologous ovine mesenchymal stem cells (MSC) were used for all experiments. Bone marrow sample were harvested from the iliac crest in sheep as described previously [26]. Briefly, animals were placed in a prone position under general intravenous anesthesia using 2% xylazine (0.1 mg/kg), ketamine (4.0 mg/kg), and midazolam (0.2 mg/kg) for harvest. The mononuclear cell fraction was separated by density gradient centrifugation and MSC were isolated from the MNC fraction by their ability to adhere to the cell culture flask. For preparation of the phantoms the desired cell numbers were determined using a Neubauer counting chamber. The cells were then centrifuged at 350×g for 5 minutes and resuspended in Dulbeccós modified Eagle Medium containing 10% fetal calf serum and 1% penicillin/streptomycin.

### Particles

VSOP-5 nm particles (Ferropharm, Berlin, Germany) were used for cell labeling. VSOP-5 nm consist of an iron oxide core of 5 nm diameter coated by a citrate monomer. As previously published the uptake of VSOP-5 nm is, depending on the concentration during the incubation, as high as 96% (at 3.0 mM VSOP concentration in the medium) and significantly higher compared to normal USPIO. Additionally VSOP-5 nm are less cytotoxic [27], [28]. Labeling of stem cells with VSOP-5 nm leads to a significant reduction of T2 relaxation [29].

### Cell Labeling

**Figure 1:**
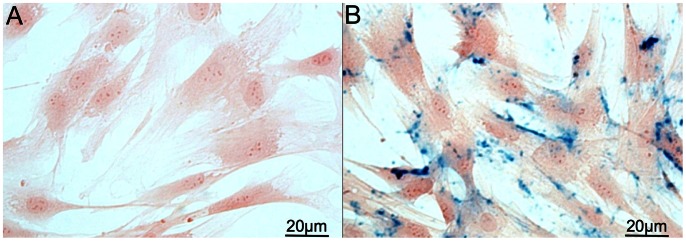
Demonstration of the VSOP uptake by the MSC. Micrographs of MSC after Prussian Blue staining after cultivation with (A) or without (B) iron oxide nanoparticles VSOP incubation (3 mM). The multiple blue dots in (B) represent VSOP particles in the cells.

VSOP-5 nm were added to the medium in a concentration of 3.0 mM and the cells were incubated for 90 min at 37°C and 5% CO_2_. No additional transfection agents were used. After incubation the cells were washed three times with PBS and were harvested. Afterwards, the cells were centrifuged at 350×g and counted. To confirm cellular iron uptake we examined cells with Prussian Blue staining (Figure 1A and 1B), relaxometry and electron microscopy, confirming a labeling efficiency of >95% of cells with this method. The reliability of VSOP labeling has also been thoroughly tested in human MSC by Heymer et al. [30].

### Agarose Phantoms

**Figure 2:**
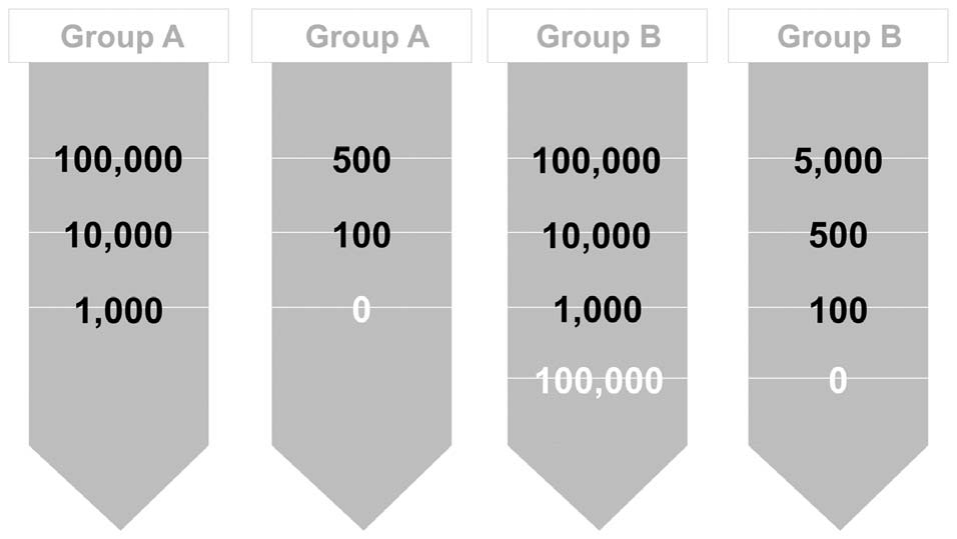
Layers of the agarose phantoms. Layer structure of group A): 2 phantoms contained layers bearing 1,000, 10,000, and 100,000 labeled MSC, and 2 phantoms comprising layers with 0, 100, and 500 labeled MSC. Layer structure of Group B): One phantom contained a layer configuration bearing 1,000, 10,000, and 100,000 labeled MSC as well as a 4^th^ layer with 100,000 non labeled MSC. The other phantom contained layers with 100, 500, and 5,000 labeled MSC as well as one more layer without MSC. Slices containing no MSC or unlabeled MSC are indicated by white numbers.

For each phantom, 20 ml agarose were filled into a 50 ml flacon, put into an ultrasound bath and consecutively centrifuged at 1500×g for one minute to remove air bubbles. For production of the cell layers 50 µL cell suspension were mixed with 50 µL of fluid agarose. The mixture of cell suspension and agarose was put into an ultrasound bath and then pipetted onto the basal layer, centrifuged at 500×g for 1 minute and consecutively cooled down. At a cross sectional area of 5.6 cm^2^ this resulted in a calculated layer thickness of approximately 17 µm. After cooling, 5 mL of agarose were pipetted onto the cell layer and centrifuged. See figure 2 for the layer structure of the phantoms.

### Magnetic Resonance Imaging

All examinations were performed in a clinical 3T MRI scanner (Magnetom Trio, Siemens, Erlangen, Germany). The phantoms where placed in the center of the used coil parallel to the z-axis of the magnetic field. Imaging parameters of the pulse sequences are listed in Table 1. SWI sequences were processed by vendor-specific software of the MRI scanner (Syngo B15, Siemens, Erlangen, Germany) and analyzed. Parameters for image contrast were set according to recommended settings [31].

**Table 1:** Sequence parameters.

MR Sequences													
No	Type	Coil	TR/TE [ms]	Voxel size [mm]	scan time [min]	Matrix	Bandwith [Hz/pixel]	Flipangle [°]	3D/2D	Slices	Averages	FOV[mm]	FOVphase[%]
A1	SWI	8ch knee	60/20	0.37×0.31×0.15	240	448	120	15	3D	160	4	140	78.1
A2	SWI	8ch knee	60/20	0.37×0.31×0.6	50	448	120	15	3D	52	4	140	78.1
A3	SWI	8ch knee	60/20	0.37×0.31×1.2	21	448	120	15	3D	40	4	140	78.1
A4	SWI	12ch head	40/20	0.39×0.31×0.7	32	256	120	15	3D	52	4	80	100
A5	T2*	12ch head	620/20	0.39×0.31×0.7	540	256	200	20	3D	48	2	80	100
A6	T2*	Loop	620/20	0.24×0.2×0.4	510	256	200	20	3D	20	6	50	100
A7	SWI	Loop	60/20	0.24×0.2×0.4	290	256	120	15	3D	30	25	50	100
B1	SWI	12ch head	60/20	0.31×0.27×0.25	406	448	120	15	3D	160	6	120	87
B2	SWI	12ch head	60/20	0.32×0.27×1.2	60	448	120	15	3D	60	3	60	87
B3	SWI	12ch head	60/20	0.56×0.49×0.25	41	448	120	15	3D	160	1	220	87
B4	SWI	12ch head	60/20	0.32×0.27×0.6	26	448	120	15	3D	80	1	120	87
B5	T2*	12ch head	620/20	0.55×0.47×0.3	629	256	200	20	3D	120	1	120	87
B6	T2*	12ch head	620/20	0.55×0.47×0.6	314	256	200	20	3D	60	1	120	87
B7	T2*	12ch head	620/20	0.55×0.47×1.2	188	256	200	20	3D	36	1	120	87
B8	T2*	12ch head	620/20	0.68×0.55×2	14	256	200	20	2D	25	3	140	100
B9	SWI	8ch knee	60/20	0.31×0.27×0.25	406	448	120	15	3D	160	6	120	87
B10	SWI	8ch knee	60/20	0.32×0.27×1.2	60	448	120	15	3D	60	3	60	87
B11	SWI	8ch knee	60/20	0.56×0.49×0.25	41	448	120	15	3D	160	1	220	87
B12	SWI	8ch knee	60/20	0.32×0.27×0.6	26	448	120	15	3D	80	1	120	87
B13	T2*	8ch knee	620/20	0.55×0.47×0.3	629	256	200	20	3D	120	1	120	87
B14	T2*	8ch knee	620/20	0.55×0.47×0.6	314	256	200	20	3D	60	1	120	87
B15	T2*	8ch knee	620/20	0.55×0.47×1.2	188	256	200	20	3D	36	1	120	87
B16	T2*	8ch knee	620/20	0.68×0.55×2	14	256	200	20	2D	25	3	140	100

The sequences are divided into groups (first column). Sequences ”A” belong to group A, (rater-based analysis). Sequences ”B” belong to group B, (ROI-based analysis). The parameters most relevant to contrast (TR, TE and Flipangle) are the same in almost all of the T2* weighted or the SWI sequences.

### Analysis Group A

Images were analyzed by 4 radiologists with 3 to 16 years of diagnostic MRI experience and blinded for MSC number, agarose phantom and sequence type. The radiologists were asked to rate whether the images showed signal alterations attributable to labeled MSC based on their experience, i.e. if labeled MSC were detectable in a given image or not, by using a four step scale: 1 “detectable”, 2 “probably detectable”, 3 “probably not detectable”, 4 “not detectable”. By calculating the mean of the values of the individual raters a score was established, hereafter referred to as the blinded rater value (BRV). For a BRV of 1 to 1.25 an image was classified as definitely containing labeled cells, for a BRV of 3.25 to 4 an image was classified as definitely not containing labeled cells. All other BRVs were interpreted as unsure. The inter-rater reliability was determined with Cohen’s Kappa. Group comparisons were performed to evaluate the influences of the sequence type, the slice thickness of the sequence and the number of labeled cells. Testing for significance was conducted using the Mann-Whitney-U test. P-values <0.05 were considered statistically significant.

### Analysis Group B

**Figure 3:**
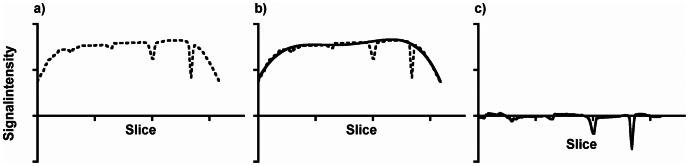
Demonstration of the fitting of the signal intensity curve to eliminate artificial effects due to sequence, gradient, and field inhomogeneities. Each value on the x-axis represents one slice of an aquired sequence. The datapoints on the x-axis therefore may vary from sequence to sequence, depending on the amount of aquired slices. The mean signal intensity per slice (as by ROI analysis) is shown for each individual slice on the y-axis, resulting in a curve with a varying resolution, depending on the amount of slices of each sequence. The original data is shown in (A). Negative peaks of signal loss indicate cell-containing layers. A signal inhomogeneity along the B_0_ axis corresponding to the long axis of the agarose phantom is superimposed, resulting in a drop of signal intensity in the first and last slices of the sequence. To remove this signal inhomogeneity without compromising cell derived peaks for further analysis a curve was fitted to the data after removal of the cell bearing slices (B) and subsequently subtracted from the original data which resulted in a straightened curve, showing the peaks on a linear baseline (C).

Images were analyzed semiquantitatively and interpreted independently. A standardized circular region of interest (ROI) of 4.0 cm^2^ was placed in the center of each acquired image and the mean signal intensity in grey values was determined. The values were imported into a statistic software (see below) for further analysis. The images of a given sequence were recorded on the x-axis in subsequent order and the corresponding signal intensities on the y-axis resulting in curves with each data point of the curve being a mean signal intensity of a specific image of a sequence. The resulting curves were fitted before further comparison to remove systemic artifacts caused by sequence, gradient, and field inhomogeneities. At first, the values of labeled MSC bearing slices were removed from the data and the remaining curve of the residuals was fitted with a fourth grade polynomial (Y = A+B*X+C*X2+D*X3+E*X4). The values of this fitted curve were subtracted from the values of the original curve, i.e. the values of the mean signal intensities including the labeled MSC bearing slices (see Figure 3). This resulted in a straightening of the curve to allow further comparison of the sequences. From this straightened curves the mean, the standard deviation, and the 0.95 confidence interval of the residuals were calculated. A significant loss of signal intensity was recorded for values being negatively outside the confidence interval.

An additional index was calculated to further analyze the data for the presence of artifacts in individual sequences, and the relationship of peaks induced by labeled cells versus peaks induced by artifacts. This “signal intensity versus artifact”-index (SIvA) is intended as a measure of the ratio between the signal intensity of labeled MSC bearing slices and the mean signal intensity of artifact related peaks. It was calculated for every peak of cell labeled slices by dividing this individual peak by the mean of all peaks caused by artifacts.

ROI placement and signal intensity readout was performed in Osirix 3.8 (Osirix, Geneva, Switzerland). All statistical analysis and all graphs were done with the software Prism 4 (Graphpad Software, La Jolla, California, USA).

## Results

**Figure 4:**
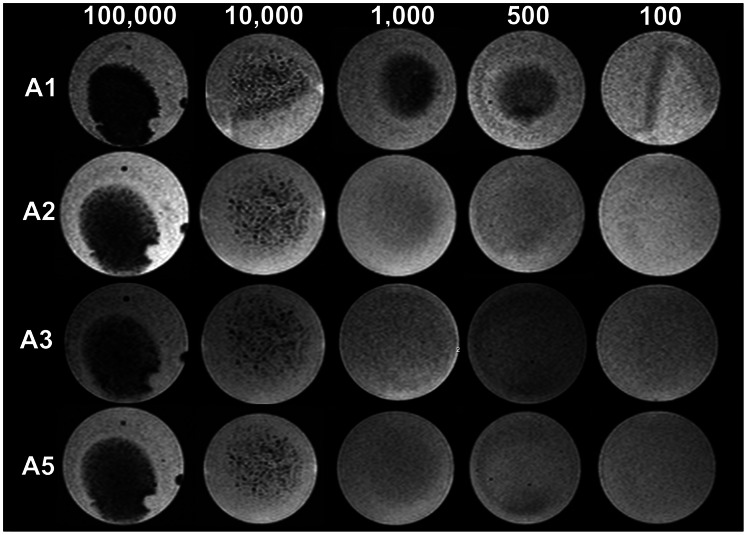
Representative phantom MR images for evaluation of the detection limit, images of Group A. Cross sectional images of results from group A at different cell concentrations and with different pulse sequences. The cell concentrations are indicated in the top row, the pulse sequences are indicated on the left according to Table 1. In sequence A1, the sequence with the highest sensitivity, a clear signal loss can bee seen at cell concentrations of 500, whereas the other sequences do not show any signal loss at this concentration. There is also some signal loss at 100 cells. Between the T2* sequence (A5) and a SWI sequence at the same voxel size (A2) no obvious difference can be seen, although the T2* weighted sequence was rated slightly more sensitive than the SWI sequence.

**Figure 5:**
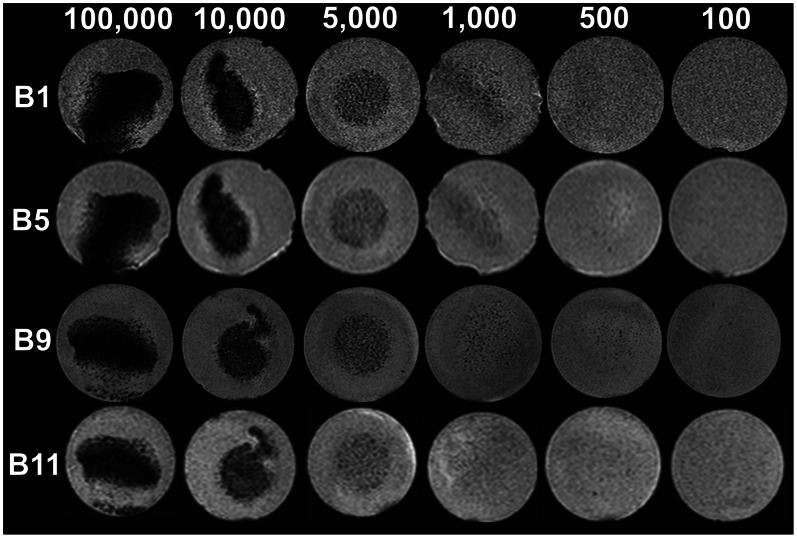
Representative phantom MR images for evaluation of the detection limit, images of Group B. Cross sectional images of results from group B at different cell concentrations and with different pulse sequences. The cell concentrations are indicated in the top row, the pulse sequences are indicated on the left according to Table 1. A diffuse signal loss at a concentration of 500 cells can be seen in B9. In B11 the signal loss at 500 cells is not obvious at inspection. Both sequences were rated sensitive for a concentration of 500 cells by ROI analysis. The higher resolution of B9 can be easily identified. The main differences between these sequences are in-plane resolution and the averages, and therefore the scanning time. Comparing B9 and B1 (8 channel knee coil and 12 channel head coil) B1 appears to be noisier although the imaging parameters remain the same. In some slices an apparently irregular shape of the container can be noted. This is due to small air inclusions at the side of the gel bloc that result in signal voids melting with the black background. The actual form, size and material of the container was the same in all phantoms.

The results of both groups are listed in Table 2, MR images of selected pulse sequences can be found in Figure 4 for results of group A and in Figure 5 for results of group B.

**Table 2:** Results of group A and group B.

VSOP labeled cells significantly detectable						
No	100000 (SivA or BRV)	10000 (SivA or BRV)	5000 (SivA or BRV)	1000 (SivA or BRV)	500 (SivA or BRV)	100 (SivA or BRV)
A1	yes (1)	yes (1)	n.a.	yes (1)	yes (1)	unsure (1.75)
A2	yes (1)	yes (1.25)	n.a.	unsure (1.75)	unsure (1.75)	unsure (1.5)
A3	yes (1)	unsure (1.5)	n.a.	unsure (2.25)	unsure (2)	unsure (2)
A4	yes (1)	unsure (1.75)	n.a.	unsure (2.75)	no (3.75)	unsure (3.5)
A5	yes (1)	yes (1)	n.a.	yes (1.25)	no (4)	no (3.75)
A6	n.a.	n.a.	n.a.	yes (1.25)	unsure (1.5)	no (3.75)
A7	n.a.	n.a.	n.a.	n.a.	n.a.	unsure (3)
B1	yes (23.55)	yes (12.73)	yes (7.73)	yes (2.33)	no	no
B2	yes (7.10)	yes (5.49)	yes (2.13)	no	no	no
B3	yes (17.11)	yes (9.74)	yes (3.38)	no	no	no
B4	yes (10.16)	yes (8.46)	yes (1.72)	no	no	no
B5	yes (16.85)	yes (10.89)	yes (1.64)	no	no	no
B6	yes (13.26)	yes (12.85)	yes (1.8)	no	no	no
B7	yes (10.36)	yes (9.67)	no	no	no	no
B8	yes (2.14)	yes (2.18)	no	no	no	no
B9	yes (16.05)	yes (13.84)	yes (7.24)	yes (4.63)	yes (3.59)	no
B10	yes (5.26)	yes (3.83)	yes (1.94)	no	No	no
B11	yes (15.86)	yes (13.12)	yes (6.39)	yes (4.13)	yes (2.61)	no
B12	yes (6.52)	yes (7.28)	no	no	No	no
B13	yes (7.29)	yes (7.61)	yes (5.52)	yes (2.48)	no	no
B14	yes (7.59)	yes (9.85)	yes (4.73)	yes (2.09)	no	no
B15	yes (5.55)	yes (5.43)	no	no	no	no
B16	yes (2.58)	yes (2.70)	no	no	no	no

The grouping is the same as in Table 1. Detectability of a certain amount of labeled MSC with a certain sequence is indicated by “yes” with the BRV (group A) or the SIvA (group B) in brackets. In group A a BRV of A or 1.25 indicates that MSC were reliably detectable. In group B MSC were recorded as detectable when the peak was outside the 0.95 confidence interval. The SIvA in group B indicates the intensity of the signal drop of a specific peak in comparison with the mean signal drop induced by artifacts. (n.a. = not applicable, this MSC quantity was not measured with this sequence). 500 labeled MSC could be detected with sequences B9, B11 and A1 with the 8 channel coil (“Knee” coil). The loop coil measurements were used as a reference only since it was not thought to be applicable in large animal scanning.

**Figure 6:**
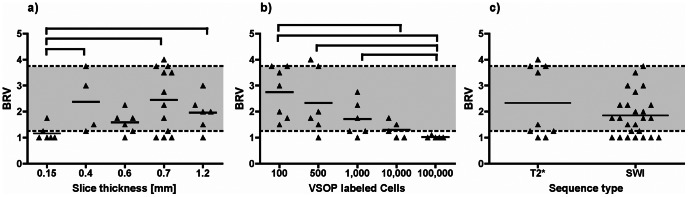
Further analysis of the rater based evaluation. Images were rated and the Blinded Rater Value (BRV) was established as described in the methods section. Significant differences (p<0.05) are indicated by brackets. The grey area between a BRV of 1.25 and 3.75 indicates BRVs regarded as “probably not detectable” or “probably detectable”. (A) Differences in BRV in relation to slice thickness of the sequence are demonstrated, regardless of other sequence parameters, sequence type or amount of labeled cells. A lower slice thickness resulted in significantly lower BRVs indicating improved detectability of cells. (B) BRVs in relation to the number of labeled cells per layer are shown. Layers with higher cell counts result in significantly lower BRVs. (C) Relationship between BRV and the imaging sequence type (SWI or T2* weighted) is shown. Differences were not statistically significant.

In group A the inter-rater reliability of the four raters was fair to good (Cohen’s Kappa 0.4–0.7) [32]. 100,000 labeled MSC could be detected in all sequences (BRV = 1). 10,000 MSC could be detected with SWI sequences using the 8 channel coil at 0.6 mm and 0.15 mm slice thickness, and with the 12 channel coil deploying a T2* weighted sequence at 0.7 mm slice thickness. 1,000 labeled MSC could be detected with the 8 channel coil with SWI at a slice thickness of 0.15 mm and with the T2* weighted sequences with both, the loop coil and the 12 channel coil, at 0.4 and 0.7 mm slice thickness. 500 labeled MSC could be detected by SWI using the 8 channel coil at 0.15 mm slice thickness. However, this sequence/coil combination also showed a false positive result in one slice without labeled MSC (data not shown). Comparing the BRVs at different slice thicknesses revealed a significantly lower BRV at 0.15 mm compared to 0.4 mm (p = 0.0381), 0.7 mm (p = 0.0429), and 1.2 mm (p = 0.0411) regardless of the sequence technique used and of the number of labeled cells (Figure 6a). Significantly lower BRVs were found for layers containing 100,000 labeled MSC in comparison to the BRVs of layers containing 1,000 (p = 0.0303), 500 (p = 0.0303), or 100 MSC (p = 0.0025). For 10,000 labeled MSC significantly lower BRVs were found in comparison to layers containing 100 labeled MSC (p = 0.0101) (Figure 6b). Comparing SWI with T2*, the differences between BRVs were not significant (Figure 6c).

**Figure 7:**
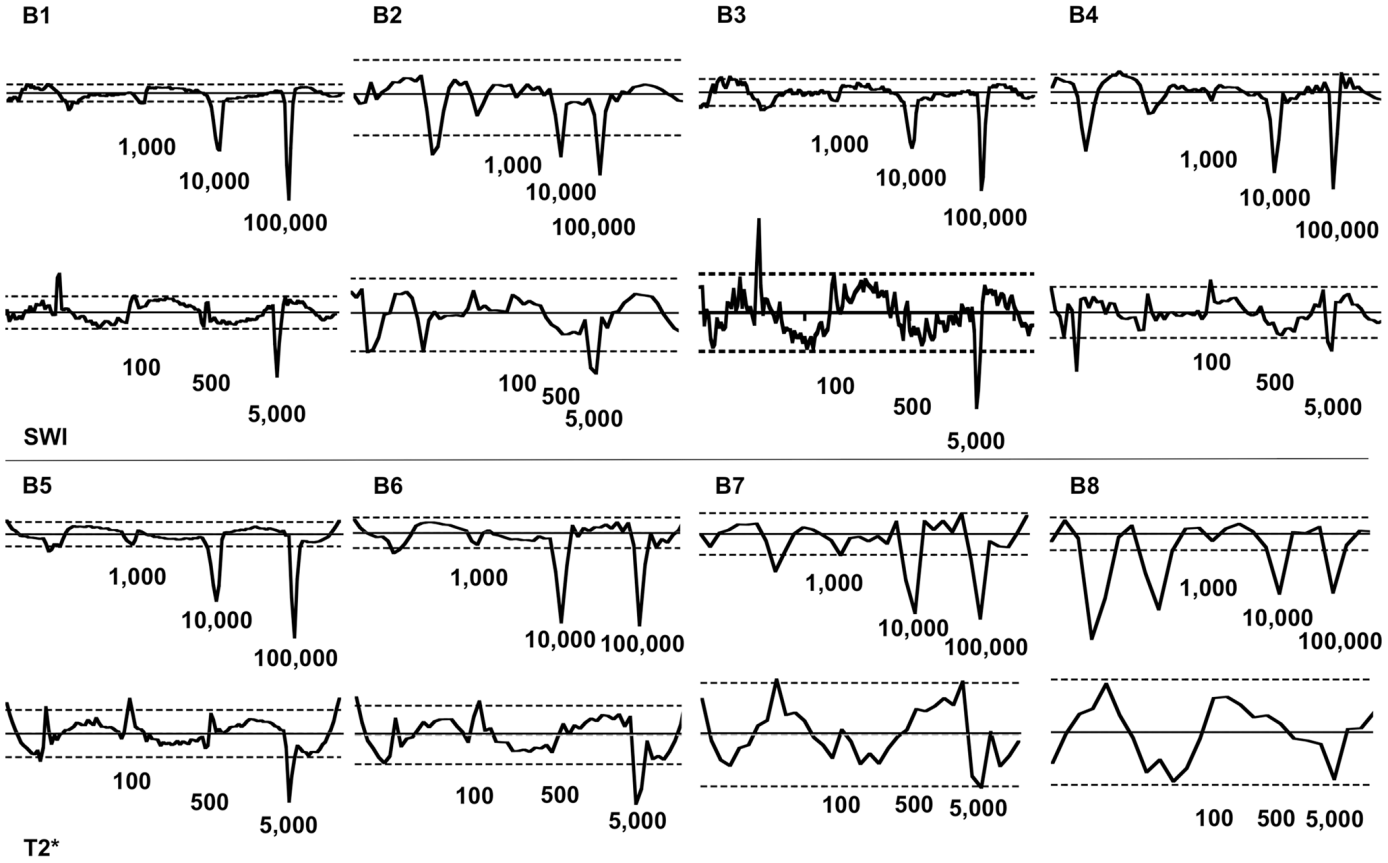
Results of the ROI based evaluation of examinations with the 12 channel coil. Results obtained by using SWI (phantoms B1 to B4) and T2* (phantoms B5 to) sequences are listed separately. According to Figure 3, on the x-axis the individual slices of the phantoms are represented consecutively, with the mean signal intensity for each slice on the y-axis (the actual axes have been removed to make the figure more clear). The dashed lines represent the 0.95 confidence interval, the straight lines represent the mean signal intensity. The amount of labeled MSC per layer is indicated for each graph in numbers below the graph. The graphs represent the changes of the mean signal intensity with each data point representing the mean signal intensity measured in a particular slice. Sequences with a lower slice thickness (i.e. better resolution of data points) depict the signal loss due to labeled MSC better than sequences with a higher slice thickness (i.e. B1 vs. B8). A similar slice thickness leads to a relatively great similarity of the performance of T2* and SWI sequences (i.e. B1 vs. B5). These graphical results are also noted quantitatively in Table 2. There are positive and negative peaks present in regions below the 100 cells layer (i.e. B2 or B5). These are artificial peaks due to inhomogeneities of the phantoms in the 0 cell layer and in the 100,000 non labeled MSC layers.

**Figure 8:**
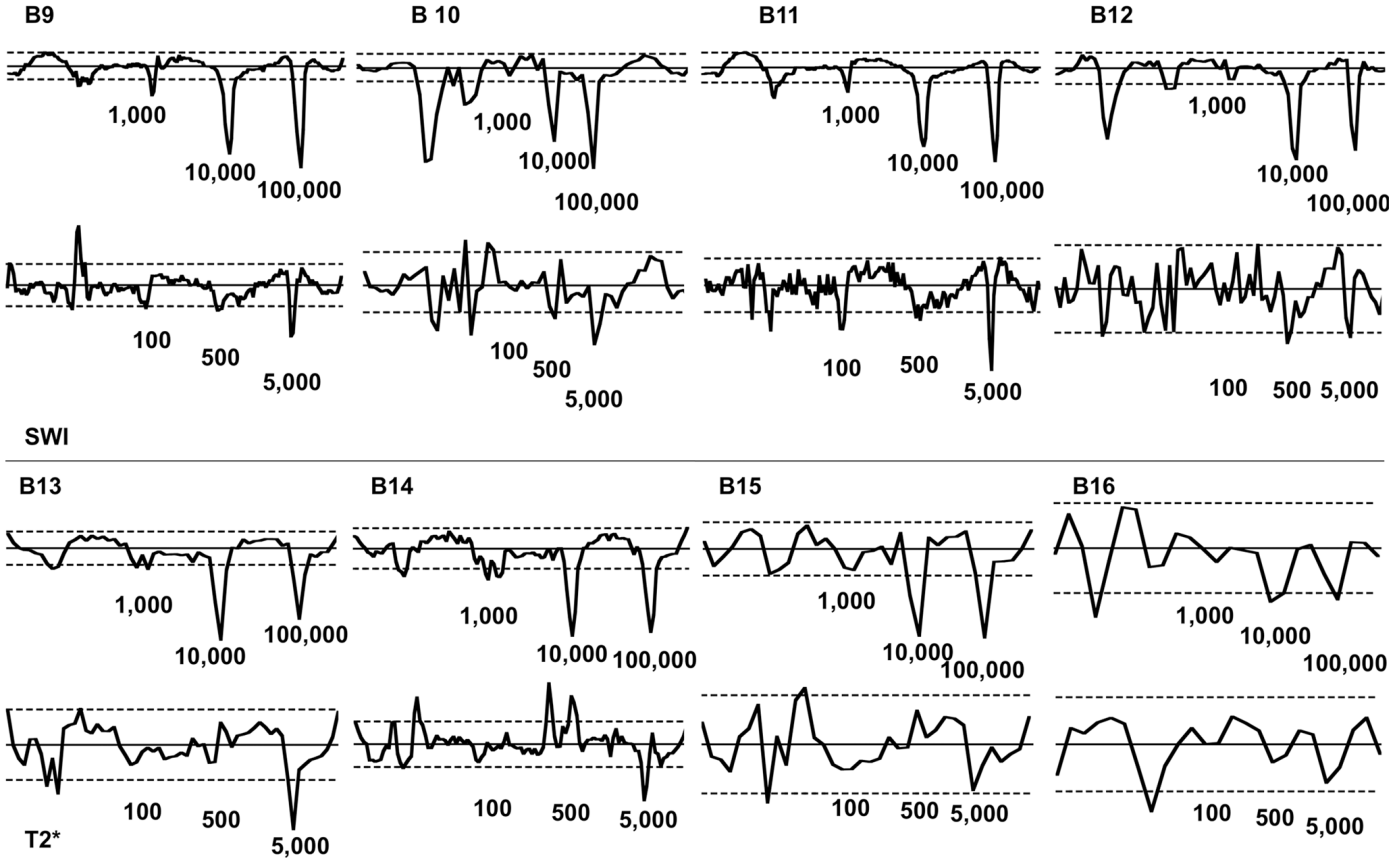
Results of the ROI based evaluation of examinations with the 8 channel coil. As in Figure 7 results obtained by using SWI (phantoms B9 to B12) and T2* (phantoms B13 to B16) sequences are listed separately. The form of the graphs is the same as in figure 7. Again sequences with a lower slice thickness (i.e. better resolution of data points) depict the signal loss due to labeled MSC better than sequences with a greater slice thickness (i.e. B11 vs. B16). With the knee coil, significant peaks at layers containing 500 labeled MSC could be identified (i.e. B9, see also table 2).

**Figure 9:**
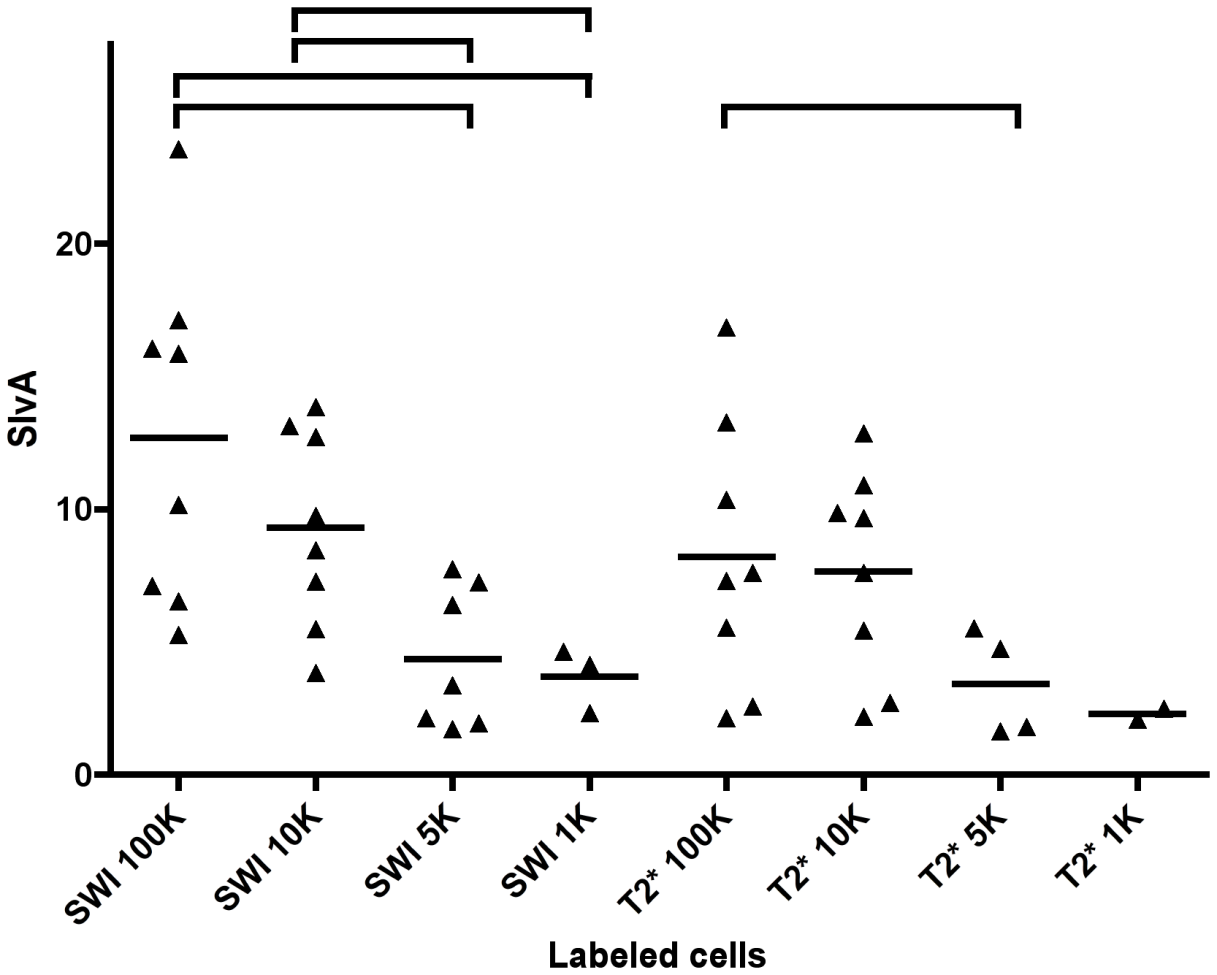
Signal intensity versus artifact-index (SivA). Statistically significant differences (p<0.05) are indicated by brackets above the data points. Larger amounts of cells per layer generate a higher SIvA indicating a better differentiation between signal drops induced by artifacts and signal drops induced by labeled cells.

In group B 100,000 labeled MSC and 10,000 labeled cells led to a significant loss of signal intensity in all sequences. 5,000 labeled MSC led to a significant signal loss in all sequences (except B7, B8, B12, B15, and B17. In layers containing 1,000 labeled MSC a significant signal loss could be measured in B1, B9, B11, B13, and B14. 500 labeled MSC could be detected with sequences B9 and B11. Graphs of the individual results of each sequence are shown in Figure 7 and Figure 8. The SIvA values (Figure 9) in SWI were significantly higher for layers containing 100,000 and 10,000 labeled MSC compared to layers containing 5,000 (p = 0.0140 and p = 0.00140) or 1,000 (p = 0.0121 and p = 0.0485) labeled MSC. T2* weighted imaging led to a significantly higher SivA in layers containing 100,000 labeled cells compared to layers containing 5,000 labeled MSC (p = 0.0485). In SWI, there was a tendency towards a higher SIvA without statistical significance.

## Discussion

By deploying two independent experimental approaches we could demonstrate that an amount of 500 VSOP labeled MSC embedded in an agarose layer phantom can be reliably detected with MRI in an experimental setup that is suitable for application in large animal models, for example in sheep. The applied SWI showed a slightly higher sensitivity than T2* imaging at a comparable spatial resolution.

MSC were labeled with VSOP belonging to the SPIO group. SPIO are the most frequently reported particles for cell labeling, mainly due to their good biocompatibility and the possibility to control their magnetic characteristics by changing the size of both the core and the cover [11]. Special characteristics of VSOP are the very good cellular uptake and the lower cytotoxicity compared to normal USPIO [28], [33]. Concerning the detectability of SPIO, as low as 500 cells could be detected in former experimental MRI examinations performed in small animal models using dedicated small animal scanners at 4.7 T to 7 T [34]–[36]. A detection limit of 100 embryonic stem cells labeled with VSOP, the same particles as used in our study, could be found after intracerebral implantation in mice with T2* weighted sequences in a dedicated small animal MRI scanner at 17.6T [29]. The same authors were able to show a linear signal loss due to accumulation of labeled stem cells at the border of cerebral infarctions in mice 48 hours after induction of the ischemia and after systemic application of VSOP labeled murine mononuclear cells in splenectomized mice at 7 T with T2* weighted sequences in a dedicated small animal scanner [17]. These detection limits are lower than the detection limit found in our study.

We identified two main reasons for this: First, the different experimental setup and the therefore lower field strength. The purpose of our examinations was the evaluation of the detection limit for VSOP labeled stem cells for the application in a large animal model. Therefore, we used a large bore clinical MRI scanner with coils applicable in larger animals but with lower field strength as in dedicated small animal scanners. Second, the length of anesthesia is crucial for the safe examination of large animals and therefore is a limiting factor. The resolution of the MR image with a signal to noise ratio still allowing for unambiguous identification of cells as determined by the field of view and the acquisition matrix is highly depending on the field strength and scanning time. However, the latter is limited and the ideal sequence for animal scanning can only be a compromise of acceptable image quality and scanning time [37]. We introduced SWI, a rarely applied sequence type for this purpose, to compensate these drawbacks. Our examinations revealed a shorter scan time with SWI for the same detection limit as compared to T2* weighted sequences. In SWI the phase information, normally discarded in T2* weighted images, is used to achieve a higher sensitivity for susceptibility induced signal changes. The sensitivity of SWI for iron deposits, microbleeds and calcifications is therefore known to be higher in comparison to T2* weighted imaging [22], [38]. However, as it is the case with T2* weighted imaging, SWI sequences cannot specifically detect iron nanoparticles. Eibofner et al. applied SWI at 1.5 T for the detection of SPIO labeled cells in agarose phantoms and in a liver model generating a positive contrast by the use of a specific image post-processing algorithm. They were able to detect about 1,000 labeled cells in an agarose phantom, which is a little less sensitve than our results [25].

Even though this study was not aimed at revealing the optimal setup of sequence parameters, one of the most sensitive SWI sequences was performed within 41 minutes (Table 1 and 2). The imaging parameters most crucial for the specific contrast (TR, TE, flip angle) were set according to the literature [31]. Therefore the main differences between the individual sequences, within the T2* or the SWI group, are voxel size and scanning time. Scanning times of up to 4 hours have been reported feasible also in spontaneously breathing sheep early after stroke induction without harming the animal [19], [26]. It may be speculated that even longer scan times are possible by using MR-compatible ventilation, allowing for a more reliable anesthesia and better control of physiological parameters. Thus, the detection limit may even be enhanced. This does not necessarily mean that scanning times of 4 h hours or beyond are recommendable, but places a scanning time of 40 min within reasonable margins. It must however be noted that the difference in the settings between SWI and T2*, especially of the difference in TR, not only influence image quality but also have a strong influence on the examination time and therefore differences in examination time apparent in this study might be reducible by further parameter optimization, especially of TR. A systemic testing of all individual parameter combinations concerning these aspects as well as their further influence, i.e. on image quality, was not performed in this study.

Agarose layer phantoms were used because they are considered more reliable than injection phantoms regarding the simulation of a possible local cell accumulation after systemic application [29]. On the other hand, layer phantoms may lead to a lower sensitivity compared with injection phantoms in terms of cell detection. Furthermore, they are prone to artifacts especially in regions of transitions between layers due to air bubbles and additional susceptibility artifacts, that could be seen in our study as well, making the identification of labeled cells more difficult.

The results of our study suggest that SWI sequences are slightly superior to T2* sequences for the detection of VSOP labeled cells in large animal models due to a a slightly higher sensitivity for labeled cells. The shorter examination time with the SWI sequences present in our study has to be interpreted with respect to the different parameter settings and might eventually be avoided by improvement of the T2* parameters although this was not systemically tested in this study. Another limitation of our examinations could result from specific artifacts of the layer phantoms not present in vivo and therefore leading to an understimation of the detection limit. On the other hand, artifacts might be present in vivo that do not exist in a layer model, for example bleedings [39].

We were able to show a detection limit for VSOP labeled stem cells that seems to be sufficient for stereotactically implanted cells, a mode of application potentially relevant for upcoming stem cell therapies for stroke [16], [40]. Further improvements of imaging sequences may decrease the detection limit to a level sufficient also for VSOP labeled stem cells after systemic injection in large animal models.

### Conclusion

Improving therapy and outcome of ischemic stroke is a highly relevant issue and stem cell therapy may become an important part of it to close the gap between acute interventions such as thrombolysis and thrombectomy, and post-stroke medical therapy. Translation of methods to large animal models and stem cell tracking by imaging will play an important role to better understand the underlying mechanisms and effects achieved. Our results demonstrate that stem cell imaging is possible and sensitive enough in a setup applicable in large animal models of ischemic stroke and stem cell therapy.
